# Intravenous tranexamic acid as an adjunct haemostat to ornipressin during open myomectomy. A randomized double blind placebo controlled trial

**DOI:** 10.1186/s13022-015-0017-y

**Published:** 2015-10-31

**Authors:** Sammy Ngichabe, Timona Obura, William Stones

**Affiliations:** Department of Obstetrics and Gynaecology, Aga Khan University, Nairobi, Kenya; Department of Obstetrics and Gynaecology, Aga Khan University, Nairobi, Kenya; School of Medicine, University of St Andrews, St Andrews, UK

**Keywords:** Haemorrhage, Myomectomy, Tranexamic acid, Ornipressin

## Abstract

**Background:**

Myomectomy is a surgical technique used for removal of uterine fibroids and historically hysterectomy has represented the mainstay of treatment. The options of conservative surgical approaches mainly aim at retention of fertility but have to be balanced against potential risks such as haemorrhage; blood loss at myomectomy still remains troublesome with use of various pharmacologic agents yielding inconclusive results. This trial aimed to explore the benefit of combining ornipressin and tranexamic acid during open myomectomy.

**Study design:**

A randomized double blind placebo controlled trial.

**Methods:**

Women who satisfied eligibility criteria were enrolled into the study and randomized into one of two groups. The experimental group received 1 g of tranexamic acid diluted to 50 ml of saline administered at 100 ml per hour at cutting time (knife to skin). The control group received placebo diluted to 50 ml of saline administered at 100 ml per hour at cutting time. Both groups had five international units ornipressin diluted in 60 ml of saline administered during surgery. The primary outcome (blood loss) was assessed by determining the weight difference of dry and soaked swabs using a digital weighing scale by converting this to volume (ml). Operating time was noted from the time of uterine incision to the time of uterine closure. The need for transfusion was determined by anaesthetists’ assessment of acceptable blood loss and clinical assessment of vital signs. Post-operative stay was calculated from the time of extubation to 8 am on the day of discharge.

**Results:**

A total of thirty-four patients were randomized to two groups; 17 received ornipressin only and 17 received tranexamic acid and ornipressin. There was no difference in blood loss between the groups with a median blood loss in the ornipressin (n = 17) and ornipressin plus tranexamic acid arms of 398 ml (IQR: 251–630) ml and 251 ml (IQR: 158–501) ml respectively P = 0.361.

**Conclusions:**

Ornipressin administered along with tranexamic acid is not beneficial for blood loss reduction at open myomectomy. In settings such as ours where myomata are prevalent and severe anaemia rampant, blood donation and judicious use of scarce blood resources is key. Efforts to optimize preoperative haemoglobin levels and blood auto-donation seem the most promising options in pre-operative preparation prior to myomectomy.

Clinical Trials Registration Number: PACTR201203000369163

## Background

Uterine myomata are the most common benign solid tumours of the female genital tract and are diagnosed in about 25–30 % of women [1]. The overall prevalence of fibroids in Black African population is estimated at 50–75 %, forming the primary diagnosis for 61 % of black and 29 % of white women [2]. Several options are available for the management of symptomatic fibroids. These can broadly be grouped into expectant, medical and surgical methods. Surgical methods include uterine fibroid embolization, laparoscopic myomectomy, hysteroscopic resection, hysterectomy or open myomectomy. Myomectomy carries a significant risk of haemorrhage with a transfusion rate of 21 % [3]. Several interventions to reduce blood loss have been explored including vasopressin analogues, antifibrinolytic agents, and gonadotropin releasing hormone analogues, oxytocin, misoprostol and mechanical tourniquets [4]. These studies have drawn conclusions from single agents versus placebo but none has utilized adjunct techniques against methods considered standard protocol. At the Aga Khan University Hospital the use of ornipressin is considered standard practice by most gynaecologists during open myomectomy and has occasionally been utilized with other additional drugs like tranexamic acid in an attempt to reduce intra operative haemorrhage. There are no randomized trials comparing adjunctive use of tranexamic acid along with ornipressin during open myomectomy and hence the need to establish its utility when used along with ornipressin.

## Methods

### Study design

This randomized double blind controlled trial compared blood loss between two groups of participants, one receiving ornipressin only and the other ornipressin and tranexamic acid. Patients were recruited from the gynaecology clinics at the Aga Khan University Hospital. Patients with documented uterine fibroids on ultrasound scan were recruited following discussion and gave written consent to participate.

The sampling frame included all women attending gynaecology clinic at the Aga Khan University Hospital. Patients with symptomatic uterine fibroids-heavy menses, pressure symptoms, and painful menses were recruited for the study. Study subjects were eligible to be enrolled into the study if they had documented symptomatic uterine fibroids by ultrasound imaging, written informed consent, no prior treatment using gonadotropin releasing hormone analogues, no family history of bleeding disorder, no current use of anticoagulants, and no prior history of deep venous thrombosis. They were excluded if they had poorly controlled hypertension or diabetes and previous uterine fibroid embolization. Sample size calculation was set at 90 % power to detect a 150 ml difference in blood loss, with a significant level of 5 % and a one sided alpha of 0.05 and beta (β)-set at 0.1.

The sample size was calculated using the formula below.

$${\text{N}} = 2\Sigma^{ 2} \left( {{\text{z}}\upalpha + {\text{Z1}}/ 2\upbeta } \right)^{2} /{\text{r}}^{2}$$

Substituting with figures:

$${\text{N}} = {{ 2 { } \times 1 3 5\times 1 3 5\left( { 1. 9 6 + 1. 2 8 2} \right)^{2} } \mathord{\left/ {\vphantom {{ 2 { } \times 1 3 5\times 1 3 5\left( { 1. 9 6 + 1. 2 8 2} \right)^{2} } { 1 50^{2} }}} \right. \kern-0pt} { 1 50^{2} }} = 1 7 {\text{ patients in each group}}.$$

Patients with documented evidence of uterine fibroids on ultrasound who had decided on open myomectomy were enrolled at the gynaecology outpatient clinic. At this point the surgical procedure informed consent was signed. Block randomization was achieved by a computer generated sequence operated by the pharmacist. The computer generated list of interventions consisted of four blocks containing a plain normal saline in 50 ml syringe and one gram of tranexamic acid diluted to 50 ml of normal saline. Random allocation to each block was made and patients allocated as per the flow of the block sequence as indicated so as to achieve allocation concealment.where T is test (Tranexamic acid) and P is Placebo (Normal Saline).

This ensured even distribution in both arms of the study [5].

The study assistant submitted the patient’s identification to the pharmacist which was entered against the intervention allocated thus keeping the randomization code unknown to the study assistant. Aseptically freshly prepared solution (50 ml syringe) of 1 g tranexamic acid in 50 ml or 50 ml normal saline (placebo) was issued to the study assistant packed in an opaque envelope and delivered to the operating room. Tranexamic acid prepared in the pharmacy is a clear solution that was diluted to 50 ml in a normal saline solution and therefore indistinguishable to the 50 ml normal saline placebo, thus assuring blinding of the surgeon, anaesthetist and data collector. The study assistant delivered the dispensed solution to theatre. The 50 ml solution loaded onto the infusion pump was started at cutting time (knife to skin) running at a rate of 100 ml per hour. All study participants enrolled received ornipressin (5 international units diluted to 60 ml) infiltrated around the myomata. Soaked swabs were weighed and blood loss estimated by subtracting the soaked weight from the dry weight and multiplied by 1.050 to convert to volume in ml [6]. The myomata location was documented post operatively by the surgeon.

Duration of surgery was calculated in minutes noted from the time of incision on the uterus to placement of the last uterine stitch, by the theatre circulating nurse. Post-operative stay was calculated from the time of extubation to 8 am on the day of discharge in hours after satisfying discharge criteria (Appendix). The volume of the myoma was determined using the Archimedes principle by immersion of all the fibroids in a measuring cylinder with water calibrated in ml. The need for transfusion was assessed by the anaesthetist if there was significant haemorrhage defined by the vital signs BP 90/60 mmHg or lower, heart rate >110 b/min and or a calculated threshold of blood loss necessitating transfusion obtained by: Hb threshold of 8 g/dl, therefore allowable blood loss was considered equal to total Blood volume (Pre-operative Hb − Hb threshold/Pre op Hb). Blood loss beyond this threshold necessitated blood transfusion. Serious adverse outcomes of the study included death, massive transfusion >5 units of blood, development of deep venous thrombosis pulmonary edema and pulmonary embolism. The study was approved by the Aga Khan University Research Ethics Committee.

## Results

**Fig. 1:**
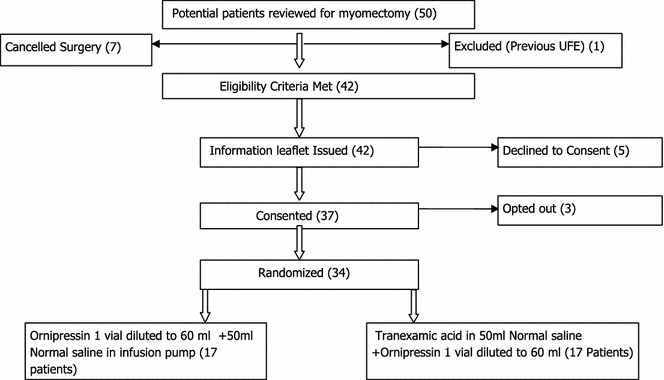
Flow of study participants

Participant flow for the study is shown in Fig. 1. Data were available for 17 participants in each group. Clinical and operative findings are presented in Table 1. Operating time and estimated blood loss are shown in Table 2. There were no major postoperative complications or study medication related adverse events in either group. Data analysis was performed using STATA version 12 special edition (STATA/SE). Categorical variables were summarized as frequencies (n) and the corresponding percentages (%) while the continuous variables that were normally distributed were summarized as mean and the corresponding standard deviation (std). Continuous variables that were skewed were transformed using the logarithm to base 10. The variables that were transformed were blood loss, operation time, and myoma volume. The test for differences between the categorical variables and the treatment group was conducted using the Pearson’s Chi Square test and because of the small cell counts for all the variables Fisher’s exact was utilized. The relationship between the continuous variables was assessed using Pearson’s correlation coefficient. The test for differences in the continuous variables between the treatment groups was done using the two-sample t test if the normality assumption was holding. However, if the normality assumption was violated, then a two sample Wilcoxon rank sum test was used. The test for differences in the continuous variables between the treatment groups was done using the two-sample t test. Assessment for the validity of a t test was also done.

**Table 1:** Demographic and baseline clinical characteristics in both groups

Variables	Group		P
	Ornipressin only (n = 17)	Tranexamic acid and ornipressin (n = 17)	
Continuous variables	Mean (std)	Mean (std)	
Age (years)	35.0 (3.9)	36.0 (4.3)	0.536
Pre operation HB	12.8 (1.3)	12.6 (1.9)	0.760
BMI (kg m^−2^)	29.9 (3.7)	29.6 (2.8)	0.775

Categorical variables	n (%)	n (%)	P
Indication for surgery			
Heavy menses	12 (71 %)	14 (82 %)	0.261
Pressure symptoms	2 (12 %)	3 (18 %)	
Subfertility	3 (18 %)	0 (0)	
Uterine incision			
Anterior vertical	9 (53 %)	8 (47 %)	1.000
Anterior transverse	2 (12 %)	2 (12 %)	
Bonney's Hood	1 (6 %)	0 (0 %)	
Posterior transverse	1 (6 %)	2 (12 %)	
Posterior vertical	4 (24 %)	5 (29 %)	
Position of myoma			
Intramural	15 (88 %)	13 (76 %)	0.656
Subserosal	2 (12 %)	4 (24 %)

**Table 2:** Outcomes in both groups

Continuous variables	Group		P
	Ornipressin only (n = 17)	Tranexamic acid and ornipressin (n = 17)	
	Mean (std)	Mean (std)	
Blood loss ^a^	398 ml (251–630) ml	251 ml (158–501) ml	0.361
Intra operative time (min) t − 0.23; *df* = 32; P = 1.000	50.1	50.1	1.000
Post-operative stay (h) (t − 1.69, P = 0.100)	67.88 (22.98)	55.03 (21.22)	0.100
Post operation HB	10.2 (2.1)	10.3 (1.9)	0.939
Surgicel use			
Yes	4 (24) %	4 (24) %	1.000
No	13 (76) %	13 (76) %	

^a^The two sample Wilcoxon rank sum test for differences in quantity of blood loss between the ornipressin and the ornipressin plus tranexamic arms showed no evidence of statistically significant difference. Data were log transformed

There was no variable that appeared to confound the true effect of the treatment. There were no missing data for all the variables.

**Fig. 2:**
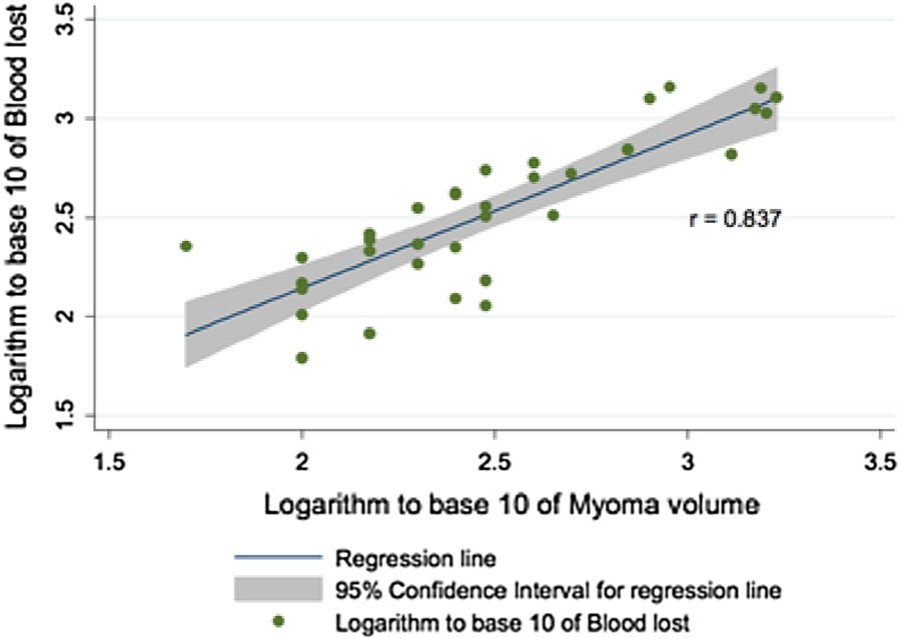
Myoma volume and blood loss

A strong statistically significant association between myoma volume and blood loss was identified (Fig. 2). Stratified by the treatment arm, the same trend was seen with correlation coefficient equal to 0.890 in the ornipressin and tranexamic acid group and 0.850 in the ornipressin group (P < 0.0001).

## Discussion

This study found that there is no additional benefit in adjunct tranexamic acid use to ornipressin during open myomectomy. This is the first study to our knowledge to investigate adjunctive use of tranexamic acid along with intramyometrial ornipressin during open myomectomy to assess for intraoperative blood loss. Open myomectomy remains a surgical option for women of low parity with large myomata desiring fertility. Haemorrhage is a possibility and use of various agents to reduce blood loss is vital. There are no studies with similar objectives to compare blood loss reduction when multiple agents are used adjunctively. Concerns of utilizing hormonal tourniquets and substances that increase uterine tone at myomectomy include concealing active bleeders that eventually become active post operatively leading to post-operative haemorrhage, not to mention other untoward effects like fever, chills and rigors with the use of misoprostol [7]. The ‘triple tourniquet’ technique has been used and showed significant results in blood loss reduction at myomectomy [8] compared to gonadotropin releasing hormone analogues. According to our findings large myomata are more likely to bleed as depicted by the correlation co efficient of 0.89 in the ornipressin plus tranexamic acid group and 0.85 in the ornipressin group. This indicates that the amount of blood loss is dependent on the volume of the myoma removed. It can therefore be insinuated that use of techniques that will reduce myomata volume pre operatively are likely to reduce blood loss at surgery. Gonadotrophin releasing hormone analogues have been utilized pre operatively with substantial reduction in myoma volume and correction of anaemia [8] however multiple challenges including cost, unfavorable side effects (menopausal symptoms, loss of bone mineral density hot flushes etc.) have limited its use. Other concerns include fibroid recurrence after cessation of gonadotrophin releasing hormone analogue use as well as difficult dissection planes encountered intraoperatively [8].

Uterine fibroid embolization offers a plausible alternative to open surgery for fibroids especially for patients not keen on surgery or whose indication for hysterectomy is due to uterine fibroids and has become a well established modality offered in our unit. A case series in Turkey compared participants who had UFE before myomectomy and those who had myomectomy alone; their conclusion was that there was significant reduction in intra operative blood loss and a 13 % need for transfusion in the latter group [9]. It is unclear whether this combined approach is really clinically useful given the additional costs of two interventions and it is not our standard practice.

One patient was excluded at recruitment who had been re admitted due to heavy menses and pressure symptoms after having uterine fibroid embolization 2 years before. The use of fibroid embolization for patients keen on fertility however remains unclear, open myomectomy still offers better  pregnancy outcomes. The Mara trial concluded spontaneous miscarriage rates were higher in patients who underwent fibroid embolization, 60 % versus 23 % in those who had open myomectomy [10]. This study population had 8.8 % of participants whose indication for surgery was subfertility.

Additional efforts pre operatively include optimizing haemoglobin levels using oral or parenteral haematinics, gonadotropin agonists and encouraging auto donation of blood to avoid risks of blood transfusion like allergic reactions, infections, transfusion associated lung injury and transfusion associated circulatory overload [11].

Uterine fibroids are more prevalent in the black population and possibly larger in volume; multiple large fibroids will ultimately require judicious surgical skill to minimize blood loss. Surgeons intending to perform myomectomies for large myoma especially in settings where patients are symptomatic for anaemia need to consider optimizing pre-operative haemoglobin and prepare blood before surgery. Transfusion may be inevitable when an initial haemoglobin level is already low and its complications likely due to multiple transfusions hence the importance of optimizing haemoglobin pre operatively. Human and financial resources remain scarce in the local setting and health policy makers need to invest in strengthening referral systems, optimizing blood donation processes and ensuring quality training of service providers who will offer patients options for correct management of fibroids even if it means conservative approaches through to uterine fibroid embolization.

The findings of this study additionally shed light on the unexplored combined use of various pharmacologic agents at myomectomy. The study was powered to detect the difference in blood loss at ninety percent power and based on this, a difference in blood loss was not observed between both groups. In future, studies of this nature should probably attempt to obtain a sample size based on how much blood loss would like to be avoided so as to recruit those who bleed excessively beyond a preset threshold. This will require a large number of participants and possibly as a multi-center study to demonstrate the true effect of the intervention on patients who are likely to bleed excessively. The study had proper randomization and blinding yielding the true effect of the intervention and both groups were similar at baseline making the results generalizable. Most Kenyan rural institutions may not have the advantage of having hormonal tourniquets due to cost, thus application of similar interventions may be hindered.

### Strengths

Randomization and blinding was achieved satisfactorily. The surgeon, principal investigator and anaesthesiologist undertaking procedure in theatre were blinded to the intervention hence eliminating potential bias in theatre. This ensured the true effect of intervention being captured which is key in studies of this nature. Secondly all patients received the intervention at the same time and same flow rate by use of an infusion pump, something similar studies have failed to standardize.

### Limitations

Surgeon’s technique at myomectomy could not be fully standardized due to various myoma positions.

### Conclusion

Adjunct tranexamic acid use to ornipressin during open myomectomy does not have any benefit in reducing blood loss. Large myoma volume notably found in the black population are likely to bleed and hence there is a need for judicious surgical skill and strategies to reduce blood loss.

## Appendix: Discharge criteria

- Patient in good general condition.
- Normal vital signs (Blood pressure, Heart Rate, Temperature).
- Second day post operative haemoglobin done and recorded in the file.
- No clinical and laboratory pointers of infection.
- Patient remains discharged from the study even if she is still in the ward but sorting out other non medical issues e.g. hospital fees, awaiting drugs.
